# Phytochemical Analysis and Anti-Inflammatory Activity of Different Ethanolic Phyto-Extracts of *Artemisia annua* L.

**DOI:** 10.3390/biom11070975

**Published:** 2021-07-02

**Authors:** Giulia Abate, Leilei Zhang, Mariachiara Pucci, Giulia Morbini, Eileen Mac Sweeney, Giuseppina Maccarinelli, Giovanni Ribaudo, Alessandra Gianoncelli, Daniela Uberti, Maurizio Memo, Luigi Lucini, Andrea Mastinu

**Affiliations:** 1 Department of Molecular and Translational Medicine, Division of Pharmacology, University of Brescia, 25123 Brescia, Italy; giulia.abate@unibs.it (G.A.); m.pucci003@unibs.it (M.P.); g.morbini001@studenti.unibs.it (G.M.); e.macsweeney@studenti.unibs.it (E.M.S.); giuseppina.maccarinelli@unibs.it (G.M.); giovanni.ribaudo@unibs.it (G.R.); alessandra.gianoncelli@unibs.it (A.G.); daniela.uberti@unibs.it (D.U.); maurizio.memo@unibs.it (M.M.); 2 Department for Sustainable Food Process, Università Cattolica del Sacro Cuore, 29122 Piacenza, Italy; leilei.zhang@unicatt.it

**Keywords:** *Artemisia annua* L., artemisinin, phenolic compounds, terpenoids, TNF-α

## Abstract

*Artemisia annua* L. (AA) has shown for many centuries important therapeutic virtues associated with the presence of artemisinin (ART). The aim of this study was to identify and quantify ART and other secondary metabolites in ethanolic extracts of AA and evaluate the biological activity in the presence of an inflammatory stimulus. In this work, after the extraction of the aerial parts of AA with different concentrations of ethanol, ART was quantified by HPLC and HPLC-MS. In addition, anthocyanins, flavanols, flavanones, flavonols, lignans, low-molecular-weight phenolics, phenolic acids, stilbenes, and terpenes were identified and semi-quantitatively determined by UHPLC-QTOF-MS untargeted metabolomics. Finally, the viability of human neuroblastoma cells (SH-SY5Y) was evaluated in the presence of the different ethanolic extracts and in the presence of lipopolysaccharide (LPS). The results show that ART is more concentrated in AA samples extracted with 90% ethanol. Regarding the other metabolites, only the anthocyanins are more concentrated in the samples extracted with 90% ethanol. Finally, ART and all AA samples showed a protective action towards the pro-inflammatory stimulus of LPS. In particular, the anti-inflammatory effect of the leaf extract of AA with 90% ethanol was also confirmed at the molecular level since a reduction in TNF-α mRNA gene expression was observed in SH-SY5Y treated with LPS.

## 1. Introduction

**Figure 1:**
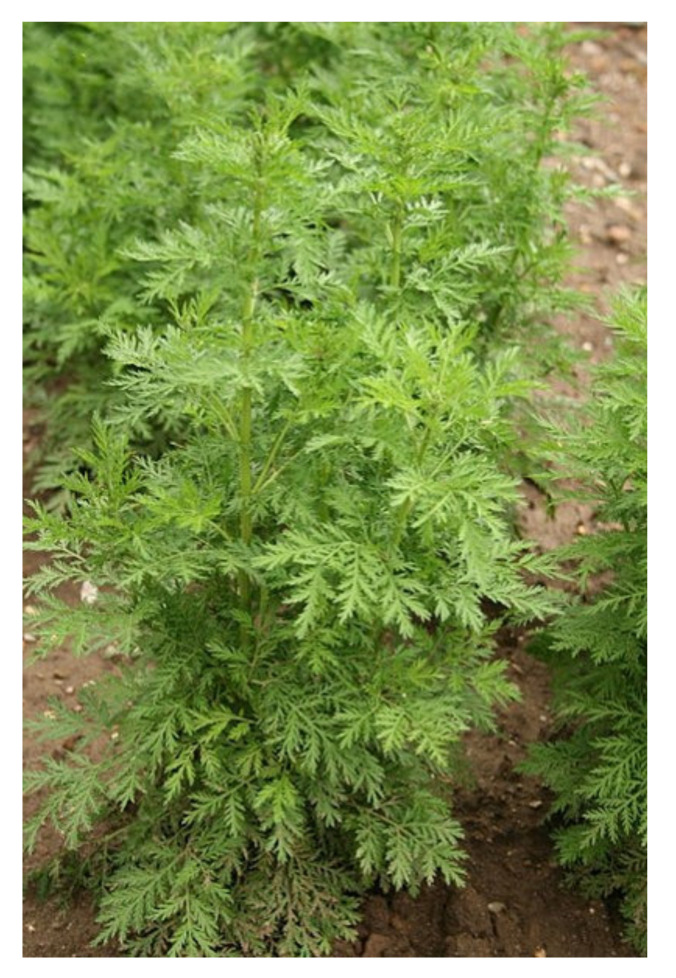
Crops of *Artemisia annua*.

*Artemisia annua* L. (AA) (Figure 1) is an annual plant native to Asia, with a herbaceous habit, belonging to the Asteraceae [1,2]. The typical habitats of AA are steppes and ecosystems at an altitude of 1000–1500 m above sea level [2]. From an organographic point of view, AA shows a single stem, which can reach 2 m in height, and has leaves that can vary from 1.5 to 6.5 cm in length [1]. Glandular trichomes can be found both in leaves and the stem of AA and be rich in biologically active secondary metabolites. More than 600 secondary metabolites have been identified in AA, including sesquiterpenes, monoterpenes, and phenolic compounds, such as coumarins and flavonoids [3]. In particular, more than thirty sesquiterpenes have been identified in AA, which are mainly located in the aerial part [3,4].

Artemisinin (ART), isolated for the first time in 1972 by the Nobel Prize winner Youyou and then structurally defined in 1979 [5], is the main sesquiterpene that, together with its derivatives, characterizes the biological action of AA. ART is a secondary metabolite synthesized and released within the glandular trichomes on the surface of the stem, leaves, and flowers of AA [5,6]. When AA reaches full maturity, leaves and flowers contain 89% of the total concentration of ART. Initially, the pharmacological studies of ART were focused on its antimalarial action. In particular, ART and its derivatives were found to be selectively absorbed by erythrocytes infected by the malarial plasmodium and subsequently localized in the membranes and mitochondria of the parasite [7]. The endoperoxide bridge present in the chemical structure of ART and its derivatives is responsible for the generation of free radicals that damage the plasmodium membrane and kill it. ART and its derivatives have been found to be effective against the various severities of malaria, especially those resistant to conventional therapies [7].

A broad and diverse phenolic profile, including different flavonoid sub-classes, caffeoylquinic acids, and derivatives of ferulic acid, has been reported for several Artemisia plant species [8,9,10]. *Artemisia annua* L., ART and its derivatives, as well as Artemisia-related phenolics have also shown additional biological activities, such as: (i) inhibition of cell proliferation; (ii) induction of cell cycle arrest and apoptosis; (iii) inhibition of inflammation and oxidative stress; and (iv) inhibition of angiogenesis, invasion, and metastasis [11]. Regarding the anti-inflammatory action, it has been observed that all AA derivatives inhibit the action of “mitogen-activated protein kinase” (MAPK), inhibit the transcription factor “nuclear factor kappa-B” (NF-κB), and inhibit the expression of the “Toll-Like Receptor” (TLR4 and TLR9) [11,12]. In addition, AA extracts in ethanol exhibit greater amounts of ART and exhibit a greater ability to block the inflammatory response induced by lipopolysaccharide (LPS) [13]. Furthermore, the phyto-extracts of AA were found to be effective in inhibiting the production of nitric oxide (NO), prostaglandins E2, and inflammatory cytokines (IL-1β and IL-6) in macrophage cultures under the pro-inflammatory stimulus elicited by LPS [13]. Another in vitro study showed that AA infusion exerts an anti-inflammatory effect reducing the release of IL-6 and IL-8 in Caco-2 intestinal cells stimulated with LPS [14]. Finally, to evaluate the toxic effects of AA and its derivatives, different studies were performed on cellular models of neuroblastoma [15,16,17].

Other secondary metabolites of AA, including rosmarinic acid, casticin and chrysosplenol D, scopoletin, chrysosplenetin, eupatorin, and β-sitosterol-glucoside, contribute to the anti-inflammatory activity [18]. Indeed, AA-isolated flavonoids also show anti-inflammatory properties both in cell cultures of mouse macrophages and in vivo on mice with local or systemic inflammation [4].

Plants produce secondary metabolites in different environmental and physiological conditions [19,20,21,22,23,24]. These secondary metabolites are responsible for the biological activity observed in numerous patho-physiological conditions. For many centuries in Asian countries, many diseases were treated with infusions and decoctions of phyto-extracts derived from AA leaves whose pharmacological bases have yet to be defined [25,26]. In commercial products, ART levels are rarely quantified. At the same time, it remains to be clarified whether differences can be found between the biological activity observed for the plant extracts and for isolated ART [27,28,29]. Thus, the aim of this study is to profile the main secondary metabolites (to include the different phenolic sub-classes and ART-related compounds) and quantify ART in different ethanolic phyto-extracts of the aerial parts of AA. Thereafter, their anti-inflammatory properties against the LPS-induced pro-inflammatory stimulus were tested in vitro to provide a comparative investigation of the action of ART individually and combined with diverse Artemisia-derived phyto-complexes.

## 2. Materials and Methods

### 2.1. Plant Material

Aerial parts of *Artemisia annua* L. (AA) were collected from ten plants growing in the greenhouses of “AMBROGIO Italia” located in Leno (45°21′52.3″ N 10°11′53.9″ E), a province of Brescia, Italy. All samples were harvested in the balsamic period and subjected to hydroalcoholic extraction. 

### 2.2. Extraction Method

In order to extract the different components of AA, 2.5 kg of aerial parts of AA were shredded and deposited into a hermetic flask and subjected to maceration with a hydroalcoholic solvent at four different ethanol concentrations (0, 25, 50, and 90%, respectively) in a total volume of 3.5 L. The extraction process of the aerial parts of AA lasted for 48 h at room temperature. One sample of each extract was stored at the Department of Molecular and Translational Medicine, Division of Pharmacology, University of Brescia.

After 48 h, the hydroalcoholic solution was centrifuged at 10,000× *g* for 10 min and was diluted to 1:10 in order to be used for the HPLC chromatographic analyses. 

### 2.3. Quantification of Artemisinin in HPLC

ART standard (10 mg) was purchased from Sigma-Aldrich (Darmstadt, Germany). The standard sample was solubilized in dimethyl sulfoxide (DMSO, 10 mg/mL) and diluted in methanol.

The separation and quantification of artemisinin (ART) were carried out using a Prominence-i HPLC instrument (Shimadzu, Kyoto, Japan) consisting of an LC-2030C binary pump, a thermostatic autosampler, and a UV detector. The column used was a Kinetex Evo C18 (Phenomenex, Torrance, CA, USA) 5 μm, 250 × 4.6 mm, 100 Å. The mobile phases were: (A) 0.1% formic acid, and (B) acetonitrile. The gradient conditions were: from 0 to 2 min, 0% solvent B; from 2 to 12 min, from 0% to 95% solvent B; from 12 to 14 min, 95% solvent B; from 14 to 15 min, from 95% to 0% solvent B; and from 15 to 20 min, 0% solvent B. The flow used was 0.6 mL/min, the column temperature was set at 30 °C, and the injection volume of all samples was 10 μL. The detection wavelength of ART was set at 254 nm. 

In order to confirm the ART peak in hydroalcoholic extracts and in the artemisinin standard, manual collections were performed in the elution time interval 14′50″ and 15′9″. These samples were lyophilized and used for the ESI-MS analysis (see below).

Once the identity of the peak was confirmed, a linear calibration curve was constructed with seven points corresponding to ART concentrations (25, 50, 100, 250, 500, 1000, and 1500 μg/mL) plotted on the *x*-axis and with their peak area at 254 nm on the *y*-axis.

### 2.4. ESI-MS Analysis of Artemisinin

Mass spectra were recorded by direct infusion electrospray ionization (ESI) on an LCQ Fleet ion trap mass spectrometer (Thermo Fisher Scientific, Waltham, MA, USA). For data processing, Qual Browser Thermo Xcalibur 4.0.27.13 software was used. ESI parameters for the sample analysis, which was performed in positive ionization mode, were as follows: spray voltage, 5.0 kV; capillary temperature, 250 °C; capillary voltage, 45 V; tube lens, 100 V. The gas flow rates (arb) were: sheath, 10; aux, 0; and sweep, 0. The infusion flow rate was set to 5 µL/min. ESI-MS analysis was performed on the hydroalcoholic phyto-extract in 90% ethanol to check for the presence of ART. An ESI-MS spectrum was acquired after the injection of a 100 µg/mL solution prepared by dissolving the lyophilized samples (hydroalcoholic extracts and artemisinin standard) of the manual collection in HPLC-DAD.

### 2.5. UHPLC-QTOF Analysis of Other Bioactive Compounds

The untargeted metabolomic analysis was performed by using liquid extracts of AA subjected to different concentrations of ethanol solvent extraction (0, 25, 50, and 90%). The samples were centrifuged at 8000× *g* × 10 min, filtered with 0.22 μm cellulose syringe filters, and then injected (6 μL) into an ultra-high performance liquid chromatography-quadrupole time-of-flight mass spectrometry (UHPLC-QTOF-MS) instrument. Specifically, we used a 1290 liquid chromatograph coupled with a G6550 mass spectrometer detector via a Dual Electrospray Jet Stream ionization system (all from Agilent Technologies, Santa Clara, CA, USA).

The instrumental conditions for the untargeted plant analysis were optimized in previous work [30]. The mass spectrometer acquisition, between 100 and 1100 m/z, was made in positive full-scan mode with a nominal resolution at 30,000 FWHM. The Agilent software Profinder B.06 was used to align and annotate the raw data according to the ‘find-by-formula’ algorithm, using a combination of the monoisotopic mass and the entire isotopic pattern, against both a phenol database (Phenol-Explorer 3.6; http://phenol-explorer.eu/, accessed date 2 July 2021) and AA-specific terpenoid compounds derived from the literature. The data were then subjected to a post-acquisition process by filtering only those compounds putatively annotated within 75% of the replications considered in at least one condition. The approach adopted corresponded to a Level 2 compound identification (i.e., putatively annotated compounds; [31]). Afterwards, compounds were ascribed to classes and subclasses and then quantified with single pure standard compounds analyzed using the same method. The standards used were representative of the following classes of bioactive compounds: anthocyanins (cyanidin), flavanols and flavonols (catechin), flavones (luteolin), phenolic acids (ferulic acid), lignans (sesamin), stilbenes (resveratrol), low-molecular-weight phenolics (tyrosols), and terpenoids (artemisinin). Results are expressed as g equivalents/kg of fresh plant material.

### 2.6. Cell Culture and Viability 

Human neuroblastoma cells (SH-SY5Y) were cultured with Ham’s F12 medium and Dulbecco modified Eagle’s medium (DMEM) in a ratio of 1:1, supplemented with fetal bovine serum (10%) and L-glutamine (0.5%), and placed in an incubator (temperature: 37 °C; 5% CO_2_) until 80% confluence was reached. Then, cells were subsequently seeded at a density of 2.5 × 10^4^ cells/mL in 96-well plates and treated for 48 h with only medium (control) or subjected to the following treatments.

In detail, SH-SY5Y cells were treated with (i) ART commercial standard (STD Artemisinin) and (ii) AA phyto-extracts at three different percentages of ethanol (25%, 50%, and 90%), where the ART concentration was quantified by HPLC. The concentrations used for the ART standard were: 50 μg/mL, 25 μg/mL, 10 μg/mL, 1 μg/mL, 500 ng/mL, 100 ng/mL, 50 ng/mL, 25 ng/mL, 10 ng/mL, and 5 ng/mL as previously reported [32]. The same concentrations were used to treat cells with AA ethanol phyto-extracts according to their ART content established by HPLC measurement.

Cell viability was assessed by colorimetric assay with 3-[4,5-dimethylthiazol-2-yl] -2,5-diphenyl-tetrazolium bromide (MTT) for the measurement of the activity of mitochondrial enzymes that reduce the MTT in formazan. Briefly, cells were incubated with 500 mg/mL of MTT for 2 h at 37 °C. Then, cells were removed and lysed with dimethyl sulfoxide. The absorbance at 595 nm was measured using a Bio-Rad 3350 microplate reader (Bio Rad Laboratories, Richmond, CA, USA).

### 2.7. Inflammatory Response and Gene Expression 

To test the anti-inflammatory action of AA phyto-extracts, different concentrations of three different AA phyto-extracts (25, 50, and 90% ethanol) and STD Artemisinin were evaluated in the presence of an inflammatory stimulus (LPS). Thus, SH-SY5Y cells were co-treated with LPS (500 ng/mL) and after 24 h cells were processed for the MTT assay as reported above.

Finally, the gene expression of tumor necrosis factor alpha (TNF-α), a pro-inflammatory gene, was investigated. In detail, cells were pre-treated with an AA phyto-extract or STD Artemisinin for 2 h and then treated with LPS for 24 h. The AA phyto-extract with the highest amount of ART was tested and the dosages protective against LPS insult were selected for both treatments.

Total RNA was then extracted from 6 × 10^5^ cells following a standard protocol [33]. The total RNA was extracted using the TRIzol^®^ Reagent (Sigma-Aldrich, Merck KGAA, Darmstadt, Germany), and two micrograms of total mRNA were reverse-transcribed using M-MLV reverse transcriptase (Promega, Madison, WI, USA).

Amplification and detection were performed with the ViiA7 Real Time PCR Detection System (Applied Biosystems, Foster City, CA, USA). The reaction mix contained 6 μL of SYBR Green Master Mix (Bio Rad Laboratories, Richmond, CA, USA), 6 pmol of each forward and reverse primer, and 2 μL of diluted cDNA. TNF-α genes were tested. The gene expression levels were normalized to the GAPDH expression level, and the data are presented as the fold change in target gene expression. The sequences of primers used were TNF-α (F: GGCAGTCAGATCATCTTCTCGAAC, R: TGGTAGGAGACGGCGATGC) and GAPDH (F: GAG TCA ACG GAT TTG GTC GT, R: TTG ATT TTG GAG GGA TCT CG).

The samples were run in triplicate, and the PCR program was initiated by 10 min at 95 °C before 40 cycles, each of which was 1 s at 95 °C and 30 s at 60 °C. Relative quantification was performed using the comparative Ct method [34]. The fold-change ratio was calculated and is expressed as mean ± SEM.

### 2.8. Chemicals

All chemicals used were of analytical grade. Ethanol (98% pure), methanol, acetonitrile, MilliQ water, and formic acid (all LCMS grade) were purchased from Merck (Darmstadt, Germany). The standards used for the UHPLC-QTOF semi-quantitative analyses were purchased from Sigma-Aldrich (Merck KGAA, Darmstadt, Germany).

### 2.9. Statistical Analysis

All experiments, analytical and on cell viability, were performed in triplicate and the data are represented as mean ± standard error of the mean (SEM). Cell viability data were analyzed by a two-way ANOVA test followed by Dunnett’s test for multiple comparisons and all *p* values < 0.05 were considered significant. All statistical analyses were performed using GraphPad Prism version 6.01 (GraphPad Software, San Diego, CA, USA). PASW Statistics 25.0 software (SPSS Inc., Segrate, Italy) was used for the analysis of variance (ANOVA; *p* < 0.05) in the semiquantitative values within each class of bioactive compounds. The Pearson’s correlation between cytotoxicity assays and different bioactive compound classes (*p* = 0.05, two-tailed) was determined with the same software.

Chemometric interpretation of bioactive compounds was performed using Mass Profiler Professional B.12.06 from Agilent (Santa Clara, CA, USA). The raw data transformation and normalization process is reported in our previous work [35]. The unsupervised hierarchical cluster analysis (HCA) was performed based on fold-change values. A supervised orthogonal projections to latent structures discriminant analysis (OPLS-DA) multivariate statistics analysis was carried out with SIMCA 16 software (Umetrics, Malmo, Sweden). Using the same software, we investigated the goodness of model parameters (goodness of fit: R^2^Y; goodness of prediction: Q^2^Y; cross-validation: CV-ANOVA, *p* < 0.01). A permutation test (n = 200) and Hotelling’s T2 test were also applied to validate and investigate outliers, respectively. Moreover, the variable importance in projection (VIP > 1.1) was selected, and we investigated the fold-change values through a pairwise comparison between each different hydroalcoholic phyto-extract and the control (0% ethanol).

## 3. Results

### 3.1. Quantification of Artemisinin in Hydroalcoholic Phyto-Extracts

**Figure 2:**
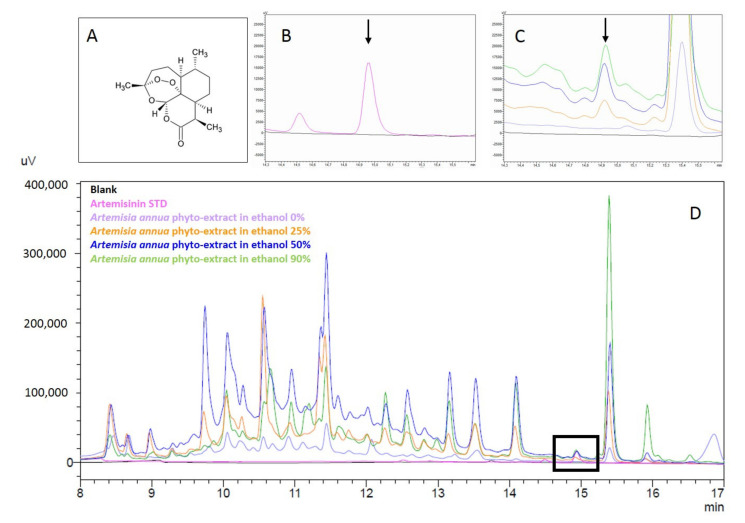
Chromatograms of the phyto-extracts of *Artemisia annua* and of the standard artemisinin. (**A**) Chemical structure of artemisinin; (**B**) details of the elution of STD artemisinin (pink) and (**C**) the ART in the AA phyto-extract samples; and (**D**) superimposed chromatograms of the ART standard and AA phyto-extracts at different concentrations of ethanol.

In order to quantify the ART in AA hydroalcoholic phyto-extracts, a linear calibration curve was constructed. The equation of the linear calibration curve was: y = 81.009x + 5033.9, with R^2^ = 0.9975. From the equation, it was possible to quantify the ART present in the various plant extracts. The chromatograms show that the STD ART eluted at 14.9 min and all phyto-extracts with 25, 50, and 90% ethanol showed a peak corresponding to the STD ART (Figure 2).

On the contrary, ART was not detected in the phyto-extracts in aqueous solution (0% ethanol). Table 1 shows the quantification of ART in the different hydroalcoholic phyto-extracts. The 90% ethanol samples were those with the highest ART levels (7.9 mg/mL), followed by the 50% and 25% ethanol samples (4.6 and 2.0 mg/mL, respectively).

### 3.2. ESI-MS Analysis of ART in the Phyto-Extracts

**Figure 3:**
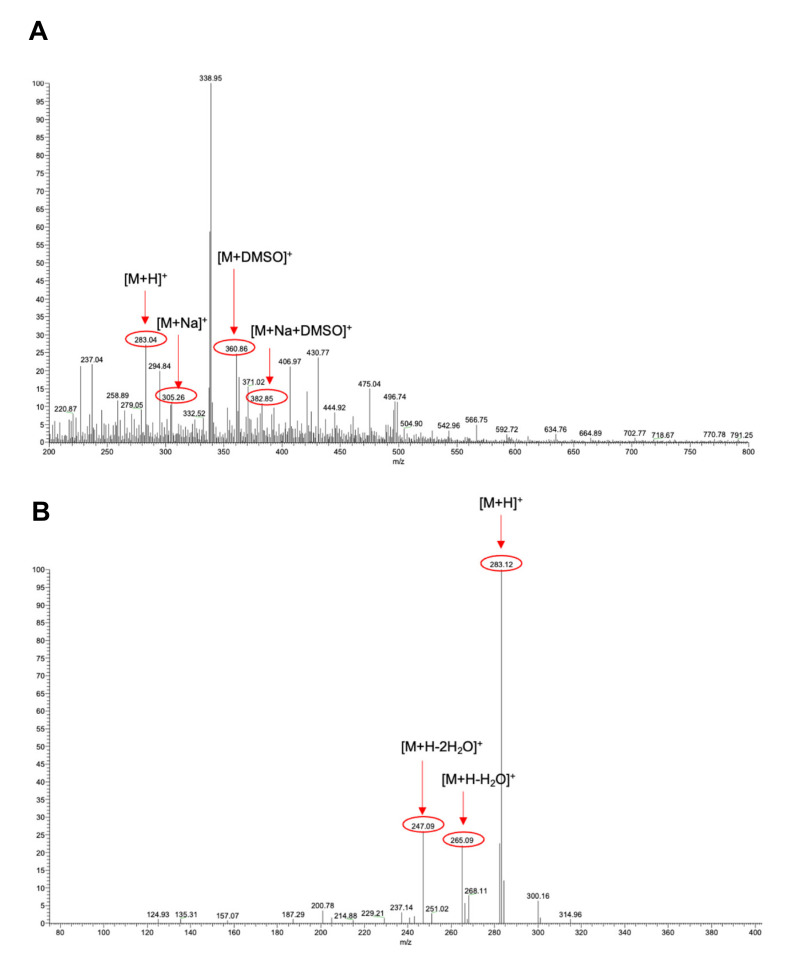
ESI-MS spectra of the sample manually collected from HPLC-DAD of the hydroalcoholic phyto-extract in 90% ethanol. (**A**) Full ESI-MS spectrum. (**B**) Fragmentation spectrum resulting from the CID experiment carried out on the isolated signal at m/z = 283.12 (normalized collision energy = 13).

ESI-MS analysis was performed on the samples obtained by manual collection performed in the elution time interval 14′50″ and 15′9″ of hydroalcoholic phyto-extracts to confirm the presence of ART. More specifically, the ESI-MS spectrum reported in Figure 3 was acquired after the injection of the sample (100 µg/mL) derived from the hydroalcoholic phyto-extract in 90% ethanol. In this sample, the signals corresponding to ART (m/z = 283.04, molecular weight = 282.33 Da, z = +1), the sodium adduct (m/z = 305.26), the DMSO adduct (m/z = 360.86), and the sodium/DMSO adduct (m/z = 382.85) were detected (Figure 3A). Moreover, other signals possibly deriving from other species from the phytocomplex are also present on the ESI-MS spectrum. At this stage, a collision-induced dissociation (CID) experiment was also performed to assess the identity of ART in the sample. The CID study carried out on the m/z = 283.12 parent ion induced the formation of two fragments with m/z values of 265.09 and 247.09 (Figure 3B). The observed fragmentation pathway was attributed to a progressive loss of water from the molecule and is in agreement with previous reports in the literature [36,37].

**Figure 4:**
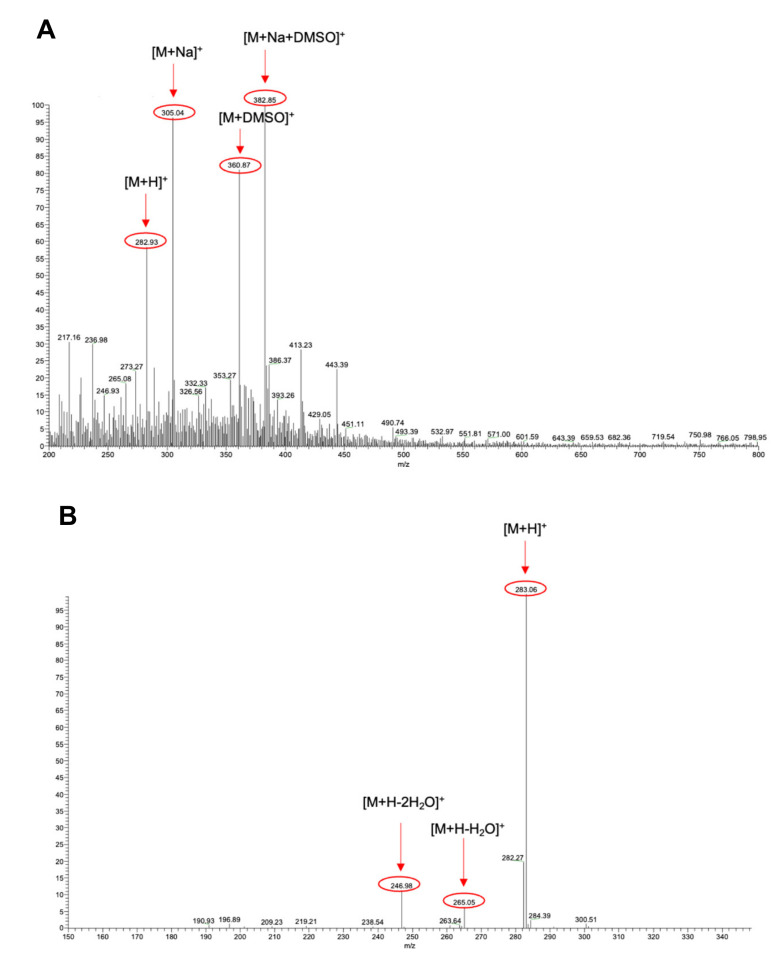
ESI-MS spectra of the sample manually collected from HPLC-DAD of the ART. (**A**) Full ESI-MS spectrum. (**B**) Fragmentation spectrum resulting from the CID experiment carried out on the isolated signal at m/z = 283.12 (normalized collision energy = 12).

Then, in order to unambiguously confirm the identity of ART in the extract, ESI-MS studies were also performed on a solution of the commercial ART standard under the same ionization conditions. The spectra reported in Figure 4 were obtained after the injection of a 10 µg/mL solution prepared by diluting the 1 mg/mL DMSO stock solution of ART in methanol. Additionally, in this case, the signals corresponding to ART (m/z = 282.93), the sodium adduct (m/z = 305.04), the DMSO adduct (m/z = 360.87), and the sodium/DMSO adduct (m/z = 382.85) were detected (Figure 4A). Consistently, results from the CID studies on the molecular ion confirmed previous results obtained from the analysis of the phyto-extract in 90% ethanol, as the same fragmentation pattern was recorded (Figure 4B).

### 3.3. Identification of Other Bioactive Compounds by UHPLC-QTOF-MS

The untargeted investigation of bioactive compounds (BCs) in hydroalcoholic phyto-extracts of AA allowed us to annotate 348 compounds (without isobaric compounds). A comprehensive list of all compounds annotated is provided in the Supplementary Materials together with classes, sub-classes, abundances, and mass spectra. In general, the main secondary metabolites found in the AA phyto-extracts were phenolic compounds (PCs), characterized by 162 flavonoids, 76 phenolic acids, 60 low-molecular-weight phenolics (LMWPs), 18 lignans, and 7 stilbenes, followed then by 25 terpenoids.

Flavonoids constitute a large group of PCs derived from the phenylpropanoid pathway. They can be further categorized into anthocyanins, flavones, flavanones and isoflavonoids, flavonols, and flavanols. The main BCs that emerged from the phytochemical profile were anthocyanins and their 3-*O*-monoglycoside or 3-5-*O*-diglycosides (i.e., cyanidin, peonidin, patuletin, and pelargonidin) and flavones conjugated with both *C*- and *O*-glycosides (i.e., luteolin, luteolin-7-*O*-glucoside, chrysosplenol D, casticin, artemetin, eupatin, apigenin, and apigenin-6,8-*C*-diglucosides) (see the Supplementary Materials). Concerning other classes of low-frequency flavonoids of AA extracts, we found flavanols, i.e., catechin, epicatechin, and epigallocatechin both alone and conjugated to the *O*-gallate group, and flavonols and their glycosides, i.e., kaempferol, quercetin, isoquercitrin, rutin, and patuletin. Regarding phenolic acids, the hydroxycinnamic acid subfamily was the most abundant and characterized by scopoletin, scoparone, 1,3-di-*O*-caffeoylquinic acid, cinnamic acid, ferulic acid, and p-coumaroylquinic acid. Moreover, we found a greater number of phenolic terpenes (i.e., carnosol, rosmanol, and carvacrol), tyrosols, curcuminoids, and alkylphenols (see the Supplementary Materials). Finally, lignans and stilbenes constitute the last two subfamilies of the PCs considered in this study. Despite their low frequencies, we found interesting compounds, such as α-conidendrin, anhydro-secoisolariciresinol, resveratrol, and piceatannol 3-*O*-glucoside. The terpenoids in AA extracts were mainly represented by sesquiterpene lactones, being artemisinin, artemisinic acid, artemisitene, deoxyartemisinin, dihydroartemisinin, arteannoides, and arteannuin B (see the Supplementary Materials).

Afterwards, all the identified BCs were semi-quantitatively analyzed using standard curves of the most representative compounds to which they belong. The results are reported in Table 1. Specifically, AA phyto-extracts were characterized by a greater source of LMWPs, lignans, phenolic acids, and terpenoids. Overall, ethanol was found to be the most effective solvent for isolating BCs. In particular, AA phyto-extracts with 50% and 90% ethanol were significantly more efficient in extracting BCs (*p* < 0.05), except for LMWPs, which were more soluble in concentrations of 25% and 50% ethanol (20.8 and 37.3 g/kg, respectively).

### 3.4. Optimal Hydroalcoholic Concentration for Bioactive Compound Extraction

In order to comparatively evaluate the different hydroalcoholic solutions used to comprehensively extract all BCs in the aerial part of AA, the metabolomics dataset was naively elaborated by unsupervised hierarchical cluster analysis (HCA; produced from the fold-change heat map normalized by median values) in order to group samples according to intrinsic similarities in their measurements (Supplementary Figure S1). The HCA showed a clear separation of the different hydroalcoholic phyto-extracts, suggesting that each solvent concentration can specifically influence the solubilization of the distinct BCs in AA plants. Accordingly, two different clusters were identified: Cluster 1 was specific to samples extracted in 0% ethanol, while Cluster 2 was composed of the samples extracted in 25, 50, and 90% ethanol. Specifically, in Cluster 2 a clear separation of these three solvent concentrations could be observed.

**Figure 5:**
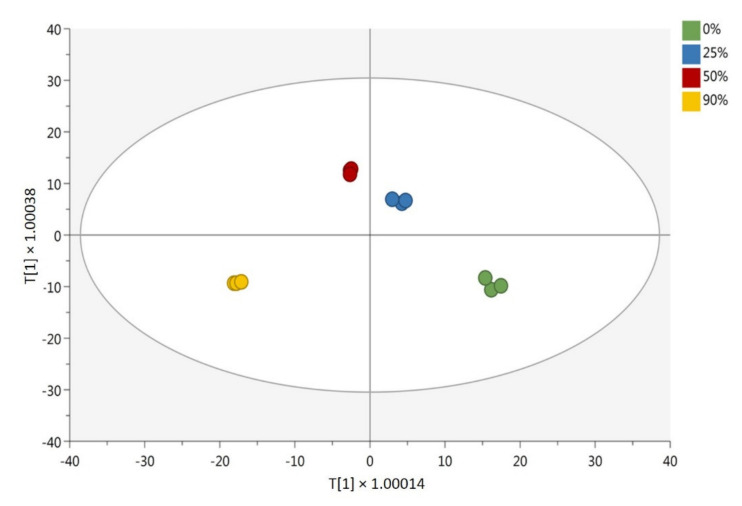
Orthogonal projection to latent structures discriminant analysis (OPLS-DA) on different concentrations of hydroalcoholic phyto-extracts of *Artemisia annua*, i.e., AA ethanol 0%, AA ethanol 25%, AA ethanol 50%, and AA ethanol 90%. The parameters: goodness of fit R^2^Y: 0.997; goodness of prediction Q^2^Y: 0.898; cross-validation ANOVA *p* < 0.01.

Afterwards, the metabolomics dataset was subjected to a further multivariate analysis, i.e., orthogonal projections to latent structures discriminant analysis (OPLS-DA), a supervised statistical tool used to identify the main discriminant biomarkers between treatments. The OPLS-DA score plot, which is reported in Figure 5, confirmed the differences in BC composition among the different phyto-extracts. In particular, the first component of the model t[1] allowed us to discriminate the phytochemical profile of AA according to the diverse polarity solvents used, namely 0% and 25% hydroalcoholic phyto-extracts (possessing a higher polarity) and 50% and 90% hydroalcoholic phyto-extracts (having a comparatively lower polarity). The second component t[2] outlined a difference in the phytochemical profile composition and concentration among different AA extracts. Indeed, the model confirmed what has been previously observed by both HCA and semi-quantitative analysis. Accordingly, each extraction solvent was characterized by distinctive phytochemical profiles. In support of our data, the OPLS-DA model built was characterized by excellent accuracy parameters, being the goodness of fit (R^2^Y) 0.997 and the goodness of prediction (Q^2^Y) 0.898. In addition, the model was cross validated and inspected for outliers (Supplementary Figure S2). The BCs were most influenced by the type of extraction method applied according to their variable importance in projection (VIP) score. Variable importance in projection (VIP) markers were selected by applying a VIP score > 1.1. The list of VIP markers, grouped into compound classes, is reported in the Supplementary Materials. The list also provides the VIP score ± standard error, the Log fold-change values (obtained by a pairwise comparison against the phyto-extract with 0% ethanol), and the indication of significance (*p* < 0.05; one-way ANOVA). As can be observed from the table, 135 compounds were found to be the most discriminative for the different extraction solvents used. In particular, VIP markers with a higher score were represented by terpenoids (i.e., artemethers, dihydroartemisinins, artemisitenes, and arteannoides), followed by lignans (α-conidendrin and isolariciresinol), LMWPs (scopoletin), and flavonoids (chrysosplenol D, (-)-Epicatechin 3-*O*-gallate, and (+)-Catechin 3-*O*-gallate), which were all upregulated in the ethanolic samples. This result suggests the importance of the solvent concentration in the recovery of BCs from the aerial part of AA.

### 3.5. Cytotoxicity Assay

**Figure 6:**
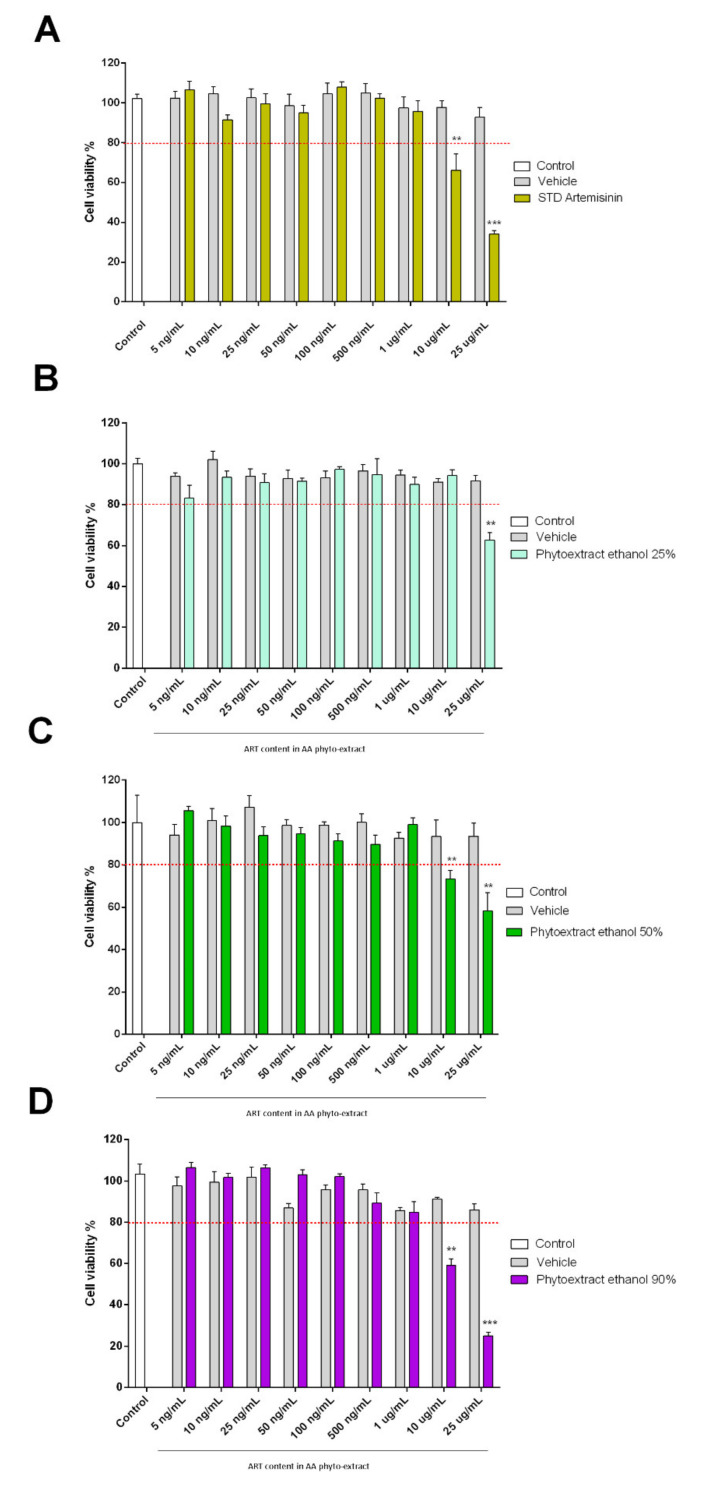
Effects of ethanolic extracts of *Artemisia annua* L. on SH-SY5Y cell viability. The bars indicate the percent cell viability of cells treated with (**A**) artemisinin standard; (**B**) phyto-extracts of AA in 25% ethanol; (**C**) phyto-extracts of AA in 50% ethanol; and (**D**) phyto-extracts of AA in 90% ethanol with the indicated concentrations (ranging from 25 μg/mL to 5 ng/mL) for 48 h and subjected to an MTT assay. Phyto-extract treatments were performed so that the ART content within the phyto-extracts was at the same concentration of STD Artemisinin. This estimation was calculated based on the phyto-extract ART quantification by HPLC. Data are representative of three replicates and shown as mean ± standard deviation; *** *p* < 0.001 vs. control group; ** *p* < 0.005 vs. control group.

In order to evaluate the biological activity of the different AA extracts, the MTT assay was performed (Figure 6). In particular, the biocompatibility of these phyto-extracts was evaluated in terms of the percentage of viable SH-SY5Y cells. Cells were treated with hydroalcoholic phyto-extracts of AA at different concentrations ranging from 25 μg/mL to 5 ng/mL for 48 h. The concentrations of the phyto-extracts used were calculated according to the ART calibration curve retrieved from HPLC analysis. The results from the MTT assay show that isolated ART shows toxicity up to 10 μg/mL (EC50 16.2 µg/mL). Furthermore, all phyto-extracts had some cytotoxic effect on SH-SY5Y cells in a concentration-dependent manner. The EC_50_ values were 36.5, 33.7, and 10.5 μg/mL for phyto-extracts at 25, 50, and 90% ethanol, respectively. All dosages above 25 μg/mL resulted in cell suffering and cell death. On the contrary, ethanolic extracts at concentrations below 1 μg/mL did not affect cell viability. The control at all concentrations relevant to the treatment group was not cytotoxic to SH-SY5Y cells. In all assays, treatments that reduced viability by more than 80% were considered cytotoxic.

### 3.6. Anti-Inflammatory Properties of Artemisinin and AA Phyto-Extracts

**Figure 7:**
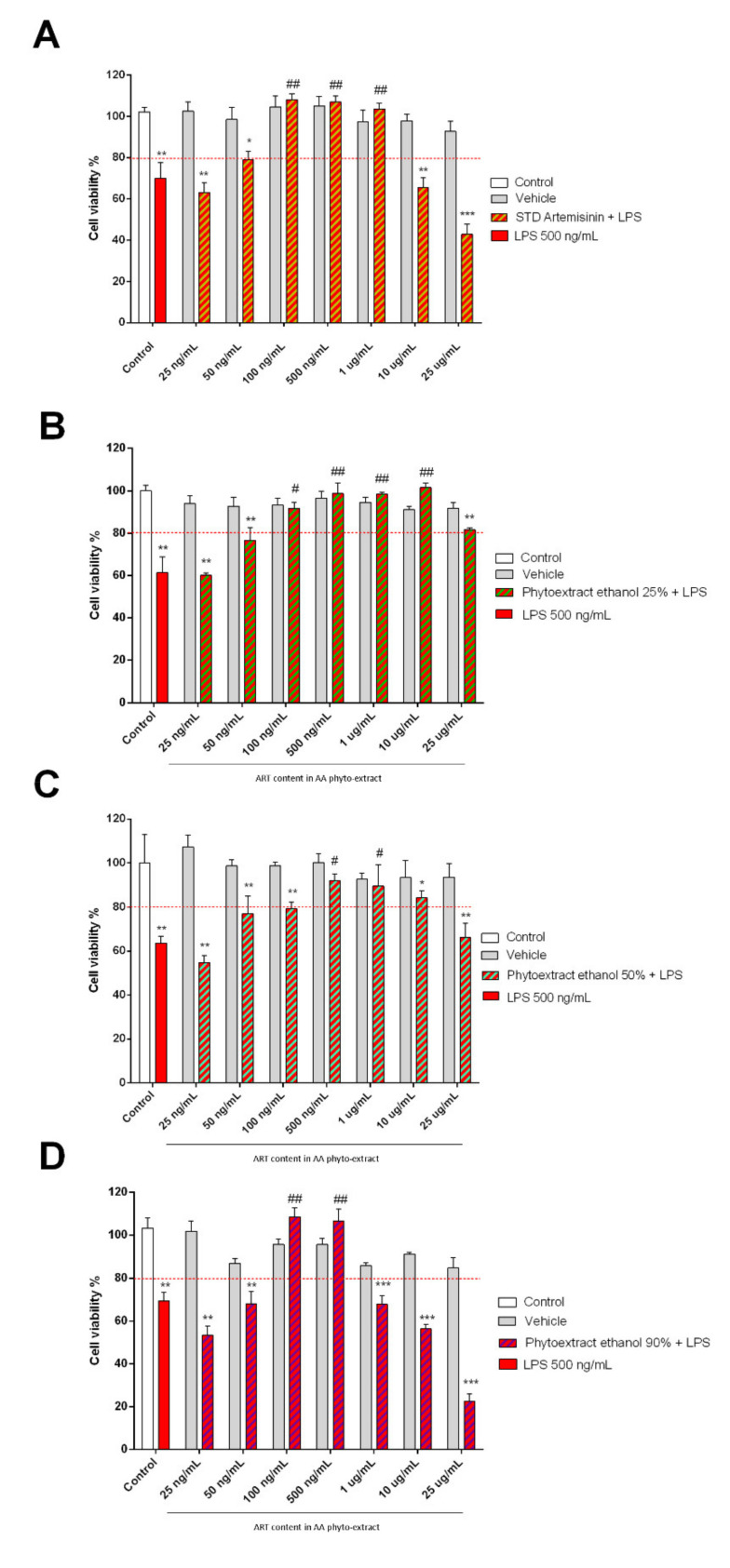
Protective effect of ethanolic extracts of *Artemisia annua* L. in SH-SY5Y cells after LPS pro-inflammatory insult. Cell viability of SH-SY5Y cells subjected to 24 h of co-treatment with LPS and Artemisinin standard (**A**), AA hydroalcoholic phyto-extract in ethanol 25% (**B**), 50% (**C**), and 90% (**D**). Phyto-extract treatments were performed so that the ART content within the phyto-extracts was at the same concentration of STD Artemisinin. This estimation was calculated based on the phyto-extract ART quantification by HPLC. Cell viability was measured with the MTT assay and is expressed as a percentage of the control (white column) that did not receive any treatment. Data are expressed as mean ± standard error of the mean: *** *p* ≤ 0.001, ** *p* ≤ 0.01 and * *p* < 0.05 versus untreated cells and ### *p* ≤ 0.001, ## *p* ≤ 0.01 and # *p* ≤ 0.05 versus LPS.

Since both *Artemisia annua* L. and artemisinin are endowed with anti-inflammatory activities, a co-treatment with LPS (500 ng/mL), an inflammatory stimulus, was performed. The LPS concentration was chosen on the basis of data previously reported in the literature [38]. As expected, the LPS treatment showed a cytotoxic effect, reducing the cell vitality by 30%. At the same time, co-treatment with ART at dosages of 1 μg/mL, 500 ng/mL, and 100 ng/mL reduced the cytoxic effect of LPS. At lower doses, ART failed to protect against the action of LPS, showing significant cytoxicity in co-treatments from 50 ng/mL to 5 ng/mL (Figure 7).

When considering results obtained from the combined treatment with hydroalcoholic AA phyto-extracts and LPS, the ethanolic extracts showed a protective action between 10 μg/mL and 100 ng/mL for the 25% ethanolic phyto-extract, between 1 μg/mL and 500 ng/mL for the 50% ethanolic phyto-extract, and between 500 ng/mL and 100 ng/mL for the 90% ethanolic phyto-extract.

**Figure 8:**
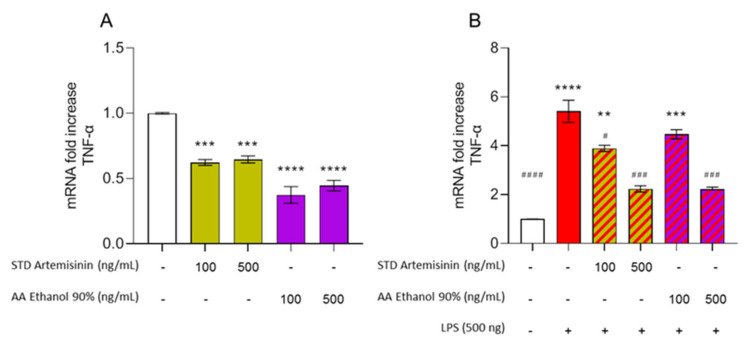
Ethanolic extracts of *Artemisia annua* and ART are able to act as a TNF-α inhibitor in the presence or absence of LPS. SH-SY5Y cells were treated for 24 h with STD Artemisinin and AA Ethanol 90% (**A**) or pre-treated for 2 h with the same treatments and then subjected to a LPS stimulus for 24 h (**B**). Cells were then processed in order to measure TNF-α m RNA levels by real-time PCR. GAPDH was used to normalize the results. Data are shown as mean ± SEM. Statistically significant differences are represented as follows: **** *p* ≤ 0.0001, *** *p* ≤ 0.001, and ** *p* ≤ 0.01 versus untreated cells and #### *p* < 0.0001, ### *p* ≤ 0.001, and # *p* ≤ 0.05 versus LPS.

Furthermore, a second treatment with LPS was performed to better dissect the molecular mechanism underlying the anti-inflammatory properties of the AA phyto-extracts. For this purpose, the mRNA gene expression of TNF-α, a potent gene-regulator of inflammatory cytokine production, was also evaluated. ART showed a significant reduction in the TNF-α mRNA gene expression at both 100 ng/mL and 500 ng/mL (*p* < 0.001). Interestingly, when the AA ethanolic phyto-extract was tested, it outperformed ART as a TNF-α inhibitor (*p* < 0.0001) (Figure 8A). On the contrary, in the presence of a pro-inflammatory stimulus (LPS treatment), the AA ethanolic phyto-extract showed a comparable performance to ART at the lowest active dosage (100 ng/mL), while at an increased dosage (500 ng/mL) only ART was able to significantly reduce TNF-α mRNA levels (Figure 8B).

### 3.7. Pearson’s Correlation between Bioactive Compounds and Cell Inflammatory Responses

Pearson’s correlation analysis was carried out in order to investigate the different degrees of correlation between the main classes of BCs found in the AA hydroalcoholic phyto-extracts and cell inflammatory responses. Specifically, we wanted to determine the relationship between the variation in BC content in different phyto-extracts and the anti-inflammatory activity detected in SH-SY5Y cell lines. The correlation table is provided in the Supplementary Materials.

Overall, a high, negative, and significant correlation between BCs and biological activity was found (on average r = 0.891; *p* < 0.01) since flavanols, flavanones, lignans, phenolic acids, stilbenes, and terpenoids showed the highest correlation. Indeed, the higher the concentration of these classes of compounds was, the lower the EC50 dosage needed to reduce the inflammation response was.

## 4. Discussion

### 4.1. Identification and Quantification of Artemisinin and Other Bioactive Compounds

One of the critical issues with using plant-derived products as therapeutic tools is the lack of quantitative information on the bioactive molecule content [39]. In particular, *Artemisia annua* L. (AA) is a plant used for therapeutic purposes in many geographical areas of the world. However, the scientific results are not always clear due to the lack of uniformity in the quantification of the active ingredients present in the phyto-extract [18]. Artemisinin (ART) is one of the biologically active molecules of AA and it is soluble in many hydroalcoholic solvents [40,41,42]. The starting point of this study was to identify and quantify ART in different hydroalcoholic phyto-extracts obtained with increasing concentrations of ethanol (25, 50, and 90%). At first, ART was initially identified in HPLC-DAD and subsequently confirmed by ESI-MS analysis. Then, ART was quantified in AA phyto-extracts. It is well-known that ART is poorly soluble in water and higher concentrations of ethanol improve its solubility in the extraction medium as observed by Martinez and colleagues [40]. So, as expected, our results show that ART has a higher concentration (7.9 ± 0.8 mg/mL) in 90% ethanol compared with the other hydroalcoholic mixtures.

The use of ethanol as a solvent also allowed us to extract other biologically active molecules, such as anthocyanins, flavanols, flavanones, flavonols, lignans, LMW phenolics, phenolic acids, stilbenes, and terpenes. In particular, the different concentrations of ethanol in the extraction solvent determined a variation in the extraction yield for the species belonging to the different chemical classes (Table 2). Anthocyanins, flavanones, and flavonols were significantly detected in 90% ethanol. LMW phenolics and stilbenes were present in the 50% ethanol. Finally, 25% ethanol showed a good extraction yield for lignans and LMW phenolics. The different extraction capacities of ethanol at different concentrations have been widely discussed in the literature [43,44,45,46].

The quantification of ART and other bioactive molecules was necessary to evaluate the biological activity at specific concentrations. In this study, the ART concentration was used to assess the viability, toxicity, and anti-inflammatory action on neuroblastoma cells.

### 4.2. Biological Activity of Phyto-Extracts

The hydroalcoholic phytochemical profiling of AA led to the annotation of a rather broad diversity of BCs, including terpenoids, flavonoids, coumarins, phenolic acids, lignans, and stilbenes. This plant is widely studied due to its biological properties, such as antimalarial, antibacterial, antiparasitic, immune-enhancing, and anti-cancer effects. Terpenes (mainly monoterpenes and sesquiterpene lactones) are the main chemical constituents of the AA phyto-extracts and have been reported to have good immunomodulatory and anti-cancer effects [47].

We detected a wide range of sesquiterpenes, including arteannuin B, artemisinin, artemisinic acid, arteannoides A–C, arteannoides I–R, deoxyartemisinin, and dihydroartemisinin, which are known for their anti-inflammatory properties. Qin and colleagues [48,49] showed the anti-inflammatory activities of terpenes isolated from dried aerial parts of AA by measuring their inhibitory effects on PGE2, NO, TNF-α, and IL-6 production in LPS-stimulated RAW 264.7 macrophages. Moreover, several phenolic compounds (PCs) with an important therapeutic activity were annotated. Such phenolics included epigallocatechin gallate, curcumin, carnosol, hydroxytyrosol, resveratrol, rosmarinic, and chlorogenic acids as potent anti-inflammatory compounds [14,50,51]. In addition to the aforementioned beneficial properties, the presence of PCs, i.e., rutin, luteolin-7-glucoside, caffeic acid, apigenin, kaempferol, and quercetin, also provide a radical scavenging ability [52].

Studies have shown that AA phyto-extracts, particularly ART and its analogues, have therapeutic effects on a variety of tumors and immune-related disorders. The mitogen-activated protein kinase (MAPK) pathway could be involved in these beneficial properties by regulating inflammatory response, cell cycle development, and apoptosis processes [47,53]. Cells are able to recognize the specific patterns of conserved molecular motifs associated with microbial pathogens (e.g., LPS) through the toll-like receptor (TLR) family, whose members are highly conserved glycoprotein receptors. This specific TLR-associated complex triggers the MyD88 downstream signaling cascade that leads to the activation of MAPK pathway signal molecules, such as I*k*B kinase (IKK), and nuclear factor-kappa B (NF-*ĸ*B) activation [54]. The anti-cancer effects of AA phyto-extracts could be attributed to the presence of casticin and chrysosplenol D, which were found in greater amounts in our samples and are able to inhibit the cell migration and pro-inflammatory cytokine production (i.e., IL-1β, IL-6, and MCP-1) induced by LPS. In particular, casticin was found to be able to inhibit I*k*Bs by blocking the phosphorylation activity of IKK and, consequently, the ĸ-*k*B cascade [55]. The same mechanism could be attributed to scopoletin, a phenolic coumarin, known for its cytotoxicity towards cancer cells, anti-inflammatory activity, and anti-angiogenesis activity [56]. NF-*ĸ*B is a transcription factor that is usually located in the cytoplasm of the cell and translocated to the nucleus when activated. The activation of NF-*ĸ*B promotes the expression of more than 400 different genes, most of which are involved in the cell regulation process, including cytokines for the cell inflammatory response, adhesion molecules, angiogenic factors (i.e., vascular cell adhesion molecule-1 (VCAM-1)) responsible for the metastatic capacity of tumor cells, and molecules that regulate the cell cycle and apoptosis, e.g., p53 factor [57,58].

Furthermore, as regards the initial exploration of the molecular mechanism underlying the anti-inflammatory properties of ART and its phyto-extracts, we can confirm that our results are in agreement with those obtained by Block and colleagues [59], who investigated ART’s anti-inflammatory effects in LPS-stimulated primary microglia.

In addition, ART was found to be effective in reducing the production of TNF-α gene expression. The synergistic effects of ART with other secondary metabolites present in the phyto-extracts strongly suggest that their natural combination can act as a potent source of TNF-α inhibitors. Again, our data are strongly encouraging since the biological activity has been confirmed at the molecular level at the lowest dosage found to be effective in LPS survival. Considering that plant-derived compounds capable of inhibiting TNF-α expression are highly attractive in the field of drug discovery [60], in future studies we will further dissect the contribution of the phytocomplex in the absence of ART (ethanol 0%). In the presence of a sub-acute pro-inflammatory status, we also confirmed that ART is a highly active compound since no differences were observed between ART and AA ethanolic phyto-extracts in modulating TNF-α gene expression after LPS treatment. Overall, these data prompt us to further explore the possible role of the AA phytocomplex in the regulation of key mediators involved in the transition from immune homeostasis to inflammation for disease prevention.

## 5. Conclusions

The initial objective of this work was to produce a set of extracts from the aerial parts of AA having different phytochemical profiles and differing in ART content. Subsequently, the concentration of bioactives in the phyto-extracts was established in a more comprehensive way using an untargeted metabolomics approach. Finally, an attempt was made to assess whether the protective action against LPS was exclusive to ART or due to the synergy with the other metabolites. This work confirmed that the highest concentration of ART was obtained with 90% ethanol. Furthermore, the relative abundance of the other secondary metabolites was found to change according to the water/ethanol ratio, given their heterogeneous chemical nature. We also determined that not all metabolites reach their maximum concentration with 90% ethanol. In particular, LMW phenolics reach their maximum abundance using 50% ethanol. Despite these differences in terms of extraction yield, the biological activity did not change. Indeed, both the ART standard and all the plant extracts showed a protective action against LPS. In addition, the AA 90% ethanol phyto-extract showed a greater ability to reduce TNF-α gene expression when used at the same dosage as ART. On the contrary, when the pro-inflammatory stimulus elicited by LPS was present, ART showed a greater anti-inflammatory response.

As some issues related to the ART-mediated response in the presence of the other secondary metabolites after an inflammatory stimulus need to be clarified, in future studies we will also explore the biological activity of the phyto-extract with 0% ethanol.

## Figures and Tables

**Table 1:** Quantification of artemisinin in extracts of *Artemisia annua* at scalar concentrations of ethanol.

Samples of *Artemisia annua*	Artemisinin (mg/mL)
Ethanol 90%	7.9 ± 0.8
Ethanol 50%	4.6 ± 0.2
Ethanol 25%	2.0 ± 0.1
Ethanol 0%	ND

**Table 2:** Semi-quantitative values of bioactive compound classes considering four hydroalcoholic phyto-extracts of *Artemisia annua* L.

Samples of *Artemisia annua*	Anthocyanins	Flavanols	Flavanones	Flavonols	Lignans	LMW Phenolics	Phenolic Acids	Stilbenes	Terpenes
**Ethanol 0%**	0.58 ± 0.05 ^a^	0.78 ± 0.03 ^a^	0.97 ± 0.06 ^a^	0.13 ± 0.01 ^a^	3.04 ± 0.17 ^a^	8.31 ± 0.36 ^a^	2.81 ± 0.12 ^a^	0.45 ± 0.04 ^a^	1.03 ± 0.10 ^a^
**Ethanol 25%**	0.97 ± 0.01 ^b^	1.10 ± 0.01 ^b^	3.13 ± 0.41 ^b^	0.13 ± 0.01 ^a^	4.07 ± 0.38 ^b^	20.81 ± 1.19 ^c^	5.39 ± 0.13 ^b^	1.38 ± 0.07 ^b^	1.25 ± 0.10 ^b^
**Ethanol 50%**	1.11 ± 0.02 ^b^	1.27 ± 0.03 ^b^	3.58 ± 0.60 ^b,c^	0.17 ± 0.05 ^a,b^	3.99 ± 0.08 ^b^	37.39 ± 1.10 ^d^	6.61 ± 0.19 ^c^	2.19 ± 0.08 ^d^	1.84 ± 0.07 ^d^
**Ethanol 90%**	2.21 ± 0.11 ^c^	1.11 ± 0.03 ^c^	4.46 ± 0.66 ^c^	0.23 ± 0.02 ^b^	4.04 ± 0.28 ^b^	11.47 ± 1.15 ^b^	6.58 ± 0.18 ^c^	1.85 ± 0.06 ^c^	1.50 ± 0.05 ^c^

Results are expressed as mean values (g equivalents/kg plant material) ± standard deviation (n = 3). Superscript letters within each column indicate the homogeneous classes that resulted from ANOVA (*p* < 0.05; Duncan’s post-hoc test).

## Data Availability

The data presented in this study are available on request from the corresponding author.

## Supplementary Materials

The following are available online at https://www.mdpi.com/article/10.3390/biom11070975/s1, Figure S1: Unsupervised hierarchical cluster analysis (HCA) of bioactive compound classes, considering four hydroalcoholic phytoextracts of *Artemisia annua* (Aa) i.e., Aa ethanol 0%, Aa ethanol 25%, Aa ethanol 50%, and Aa ethanol 90%. The model was performed using foldchange values normalized by median. Figure S2: Validation of OPLS-DA discriminant model of bioactive compound classes detected in four hydroalcoholic phytoextracts of *Artemisia annua* (Aa); Hotelling’s T2 using 95% and 99% confidence limits is given in upper pane [A], whereas outcome of permutation test (N = 100) is given in the lower one [B].
