# Supplementary material for: Phytochemical Analysis and Anti-Inflammatory Activity of Different Ethanolic Phyto-Extracts of Artemisia annua L
Source: Biomolecules. 2021 Jul 2;11(7):975. doi: 10.3390/biom11070975 (PMC8301839; doi:10.3390/biom11070975)
Supplement: Supplementary file 1 [file biomolecules-11-00975-s001.zip › biomolecules-1262537-supplement.pdf]

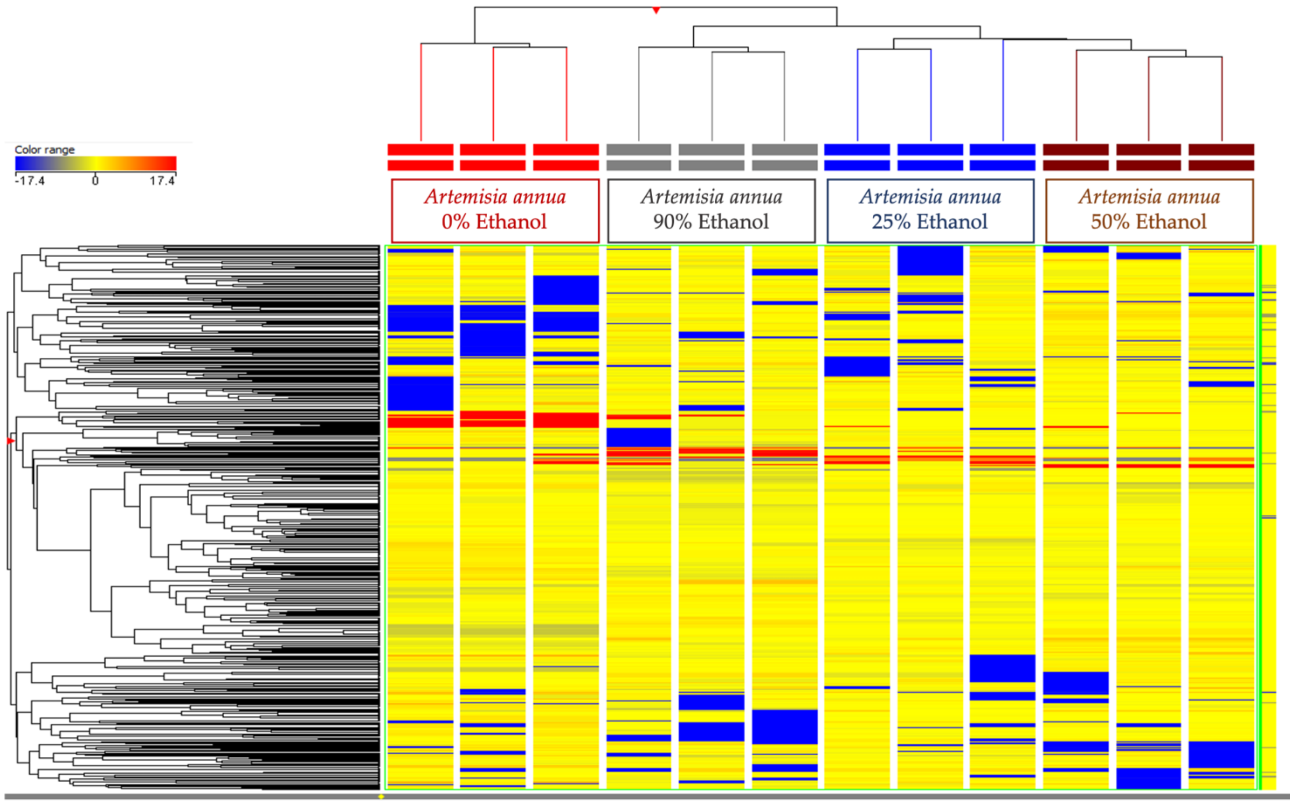


**Figure S1.** Unsupervised hierarchical cluster analysis (HCA) of bioactive compound classes, considering four hydroalcoholic phytoextracts of *Artemisia annua* (Aa) i.e., Aa ethanol 0%, Aa ethanol 25%, Aa ethanol 50%, and Aa ethanol 90%. The model was performed using foldchange values normalized by median.

A

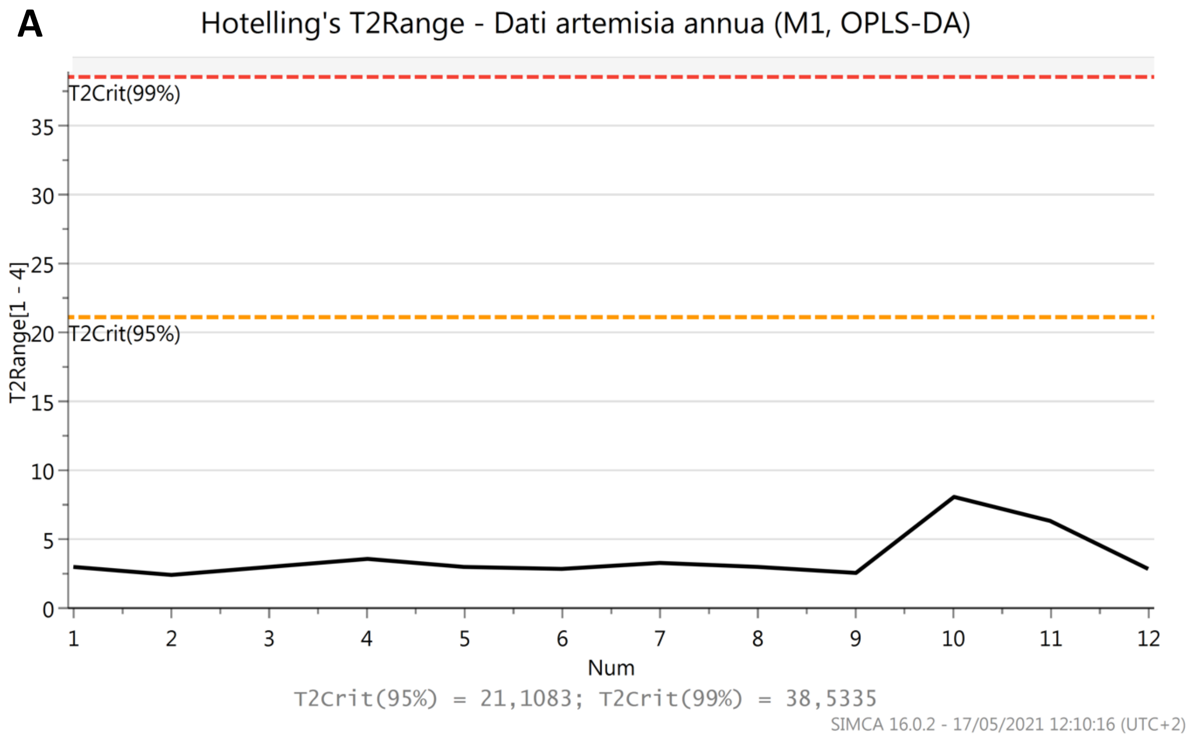

**B**

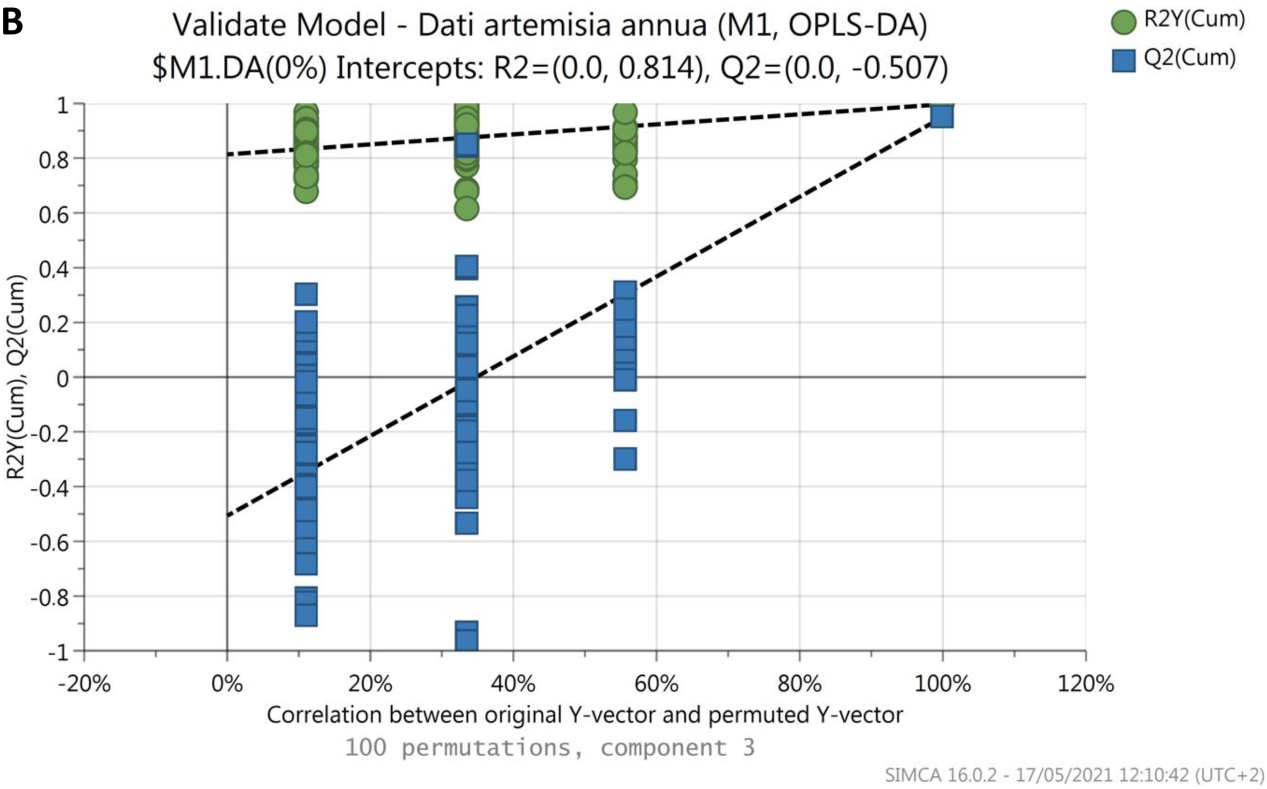


**Figure S2.** Validation of OPLS-DA discriminant model of bioactive compound classes detected in four hydroalcoholic phytoextracts of *Artemisia annua* (Aa); Hotelling's T2 using 95% and 99% confidence limits is given in upper pane [**A**], whereas outcome of permutation test (N = 100) is given in the lower one [**B**].
